# Individual and systems-related factors associated with heart failure self-care: a systematic review

**DOI:** 10.1186/s12912-023-01689-9

**Published:** 2024-02-09

**Authors:** Carolyn Kleman, Stephanie Turrise, Heidi Winslow, Omar Alzaghari, Barbara J. Lutz

**Affiliations:** 1 College of Health & Human Services School of Nursing, University of North Carolina Wilmington, 601 South College Road, Wilmington, NC 28403 USA; 2 Manager of Nurse Residencies, Novant New Hanover Regional Medical Center, 2131 S. 17th Street, Wilmington, NC 28401 USA

**Keywords:** Self-care, Heart failure, Descriptive, Systematic review

## Abstract

**Background:**

Heart failure (HF) is a prevalent condition worldwide. HF self-care is a set of behaviors necessary for improving patient outcomes. This study aims to review and summarize the individual and system-related factors associated with HF self-care published in the last seven years (Jan 2015 – Dec 2021) using the Socioecological Model as a review framework.

**Methods:**

An experienced nursing librarian assisted authors in literature searches of CINAHL Plus with Full Text, Ovid Nursing, PsychINFO, and PubMed databases for peer-reviewed descriptive studies. Inclusion criteria were HF sample with self-care as the outcome variable, and a quantitative descriptive design describing individual and/or system-level factors associated with self-care. Exclusion criteria were interventional or qualitative studies, reviews, published before 2015, non-English, and only one self-care behavior as the outcome variable. The search yielded 1,649 articles. Duplicates were removed, 710 articles were screened, and 90 were included in the full-text review.

**Results:**

A subset of 52 articles met inclusion and exclusion criteria. Study quality was evaluated using modified STROBE criteria. Study findings were quantitated and displayed based on socioecological levels. Self-care confidence, HF knowledge, education level, health literacy, social support, age, depressive symptoms, and cognitive dysfunction were the most frequently cited variables associated with self-care. Most factors measured were at the individual level of the Socioecological Model. There were some factors measured at the microsystem level and none measured at the exosystem or macrosystem level.

**Conclusion:**

Researchers need to balance the investigation of individual behaviors that are associated with HF self-care with system-level factors that may be associated with self-care to better address health disparities and inequity.

**Supplementary Information:**

The online version contains supplementary material available at 10.1186/s12912-023-01689-9.

## Background

Heart failure (HF) is a complex, progressive condition affecting 6.2 million Americans [1] with a global prevalence of 64.34 million individuals [2]. Morbidity and mortality secondary to HF remain high despite improved treatments [3]. Evidence shows that people with HF have difficulties with self-care [4, 5]. Self-care is a key component in the prevention and management of HF [6], is included in HF guidelines as a class I intervention [7], and is essential to successful long-term management. Improved outcomes, such as decreased morbidity and mortality and decreased HF hospitalizations are related to effective self-care [8, 9].

Successful HF self-care depends not only on the person with HF but also on persons and things outside of the individual. Individual input, such as self-care, *and* system-related contributors are needed for health outcome improvement. A systematic review of literature that reports both individual and systems-level factors associated with HF self-care can help describe past research, inform future research efforts, and contribute to updating theories in self-care. Conducting a systematic review of literature that collects, integrates, analyzes quality, and presents findings across many research studies provides a robust and organized method of summarizing current literature. There have been previous reviews related to HF self-care. Barnason and colleagues' [10] integrative review of 19 intervention studies (from 2000–2010) that promoted self-care in patients with heart failure in 2011 found that most interventions were cognitive-behavioral in design. These counseling and peer-support interventions improved self-efficacy. Providing HF education was helpful but as an intervention, but was not statistically significant. Oosterom-Calo et al. [11] conducted a systematic review of the determinants of HF self-care in 2012 which included 26 studies. They outlined some elements influencing self-care, such as the length of time since the patient’s diagnosis with HF, perceived benefits and barriers (as they pertain to sodium restriction), and patients with type D personality. They reported that most other determinants had inconsistent and insufficient evidence. Another systematic review looking at the determinants of effective HF self-care considered both patients’ and caregivers’ perceptions. This study by Clark et al. [12] included 49 studies (from 1995–2012) and was a review of qualitative literature whose purpose was to make recommendations for providers to help patients and caregivers increase the effectiveness of their self-care. The most recent review in 2018 was integrative and included 20 quantitative, qualitative, and mixed methods studies. This review found that increasing age, lower self-care confidence, multimorbidity, disease severity, and cognitive impairment were associated with poor self-care. Knowledge of symptom management and treatment regimen had a positive influence on HF self-care. These authors noted that the studies they included (from 2008 to 2015) did not examine environmental aspects such as cultural or ethnic influence on HF self-care and identified this as an important area for future research to consider [13]. There are no systematic reviews, within the last 5–7 years, that create a comprehensive, quality description of both the studies that have investigated individual factors and system-related factors impacting HF self-care. The current review included articles from 2015–2022 to continue where other reviews ended and to correspond with the 2016 update of the Situation Specific Theory of Heart Failure Self-care [14]. In this article, we report the results of a systematic literature review that examined the evidence regarding individual and system-related factors associated with HF self-care, determined how these factors may impact an individual's ability to engage in HF self-care, used the socioecological model to organize factors, and make recommendations for interventions to address these factors to improve and promote self-care behaviors.

### Self-care as defined in the literature

Self-care in HF is defined in multiple ways in the literature and is often used synonymously with self-management. However, Riegel and colleagues [14] include self-care management as one of three processes that comprise the construct of HF self-care: maintenance, symptom perception, and management [14]. Therefore, in their definition, HF self-care is "a naturalistic decision-making process that influences actions that maintain physiologic stability, facilitate the perception of symptoms, and direct the management of those symptoms" [14]. Self-care maintenance is defined as adhering to treatment and engaging in the recommended health behaviors, such as adhering to medication regimens, exercising, and following a low-sodium diet. Symptom perception is detecting physical sensations, such as shortness of breath or lower extremity edema, and interpreting what those sensations mean. Self-care management is the response or action to the sensations and attributed meaning of the sensations. For instance, if one attributes shortness of breath to lung disease, the individual may choose to use a rescue inhaler, but if they attribute the shortness of breath to HF, they may take an extra dose of diuretic [14]. The Theory of Situation-Specific Heart Failure Self-care considers three categories of factors that can impact self-care: (1) person, (2) problem, and (3) environment [14].

Alternatively, Moser and Watkins [15] defined HF self-care as a multidimensional life course model. In their definition, Moser and Watkins [15] are consistent with Riegel et al.'s (2016) definition of a naturalistic decision-making process. However, the factors that are associated with maintenance are somewhat different. In Moser and Watkins’ [15] definition, the term adherence is used in place of maintenance. It is a dynamic process associated with personal factors such as age, life experiences, and healthcare system experiences. Overall, there are five factors that Moser and Watkins [15] state influence self-care decision-making; 1. health literacy, 2. psychosocial status; 3. current symptoms; 4. aging and related changes such as cognitive status and comorbidities, and lastly, 5. prior experiences with symptoms and the health care system. While there is some overlap in these definitions of self-care, there are significant differences in how it is conceptualized, the factors that influence it, and the relationships among them.

Another point of conceptual confusion is that HF self-care has a different meaning than general self-care. The World Health Organization (WHO) [16] defines self-care as "the ability of individuals, families, and communities to promote health, prevent disease, maintain health, and to cope with illness and disability with or without the support of a healthcare provider." (para 1). While there are similarities, self-care in HF is more focused on the behaviors used to cope with and manage the chronic nature of HF and the variability of HF symptoms with a goal of early detection of symptom exacerbation and optimizing individual health outcomes. For the purposes of this review HF self-care is a set of behaviors necessary for improving HF-related patient outcomes.

### Theory/framework

**Fig. 1:**
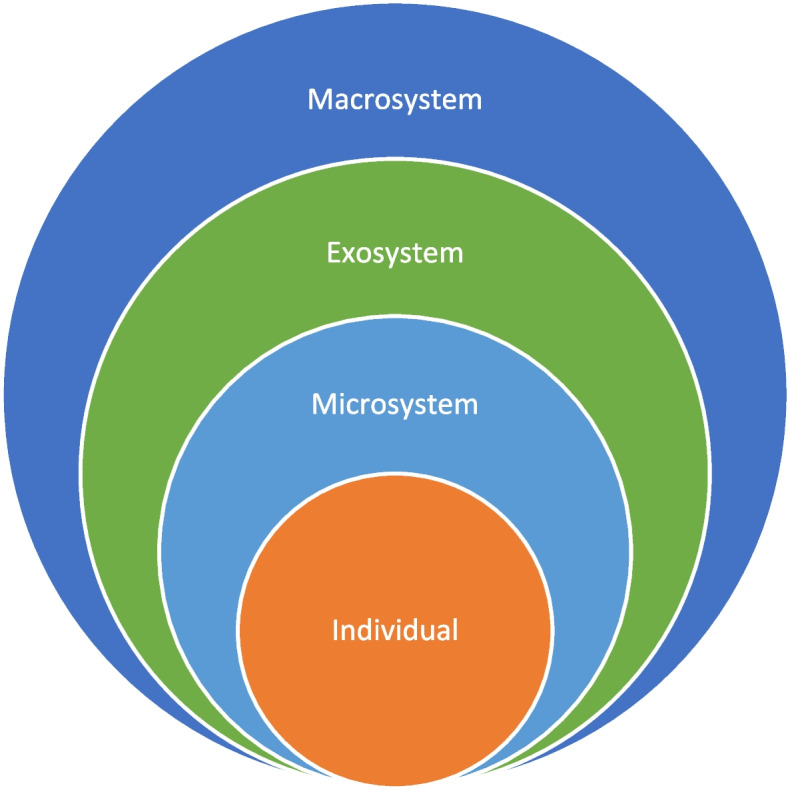
Adapted socioecological model Adapted from Bronfenbrenner, U. (1996). The ecology of human development: Experiments by nature and design. Harvard University Press

This review is guided by Bonfenbrenner's Socioecological Model [17]. The model considers levels of interaction between individuals; family, friends, neighborhood (microsystem); workplace, community-based resources, mass media, government systems, and local industry (exosystem); economic, social, educational, religious, and political systems, cultural norms, values, and ideologies (macrosystem). Interactions between systems are bidirectional. The original model includes a mesosystem representing interactions between individuals and the microsystem. Before interactions between systems can be studied, becoming familiar with and measuring components of each socioecological level is helpful. Therefore, the focus of this study was to report on the research occurring at each level, not the interactions between levels; thus, the mesosystem has been removed and the model has been adapted. See Fig. 1. In the current healthcare environment, there is an emphasis on social determinants of health and health equity. The aims of this study were to review the state of the science and identify gaps at different levels of the SEM to provide a guide for researchers and clinicians to think more holistically about self-care in HF.

## Methods

A systematic review of quantitative descriptive studies assessing self-care in HF was conducted using The Preferred Reporting Items for Systematic Reviews and Meta-Analysis (PRISMA) guidelines [18]. A university librarian helped develop the search protocol.

### Eligibility criteria

Study inclusion criteria were HF sample with self-care as the outcome variable of the study, quantitative descriptive design, and describes individual and/or system-level (microsystem, exosystem, and macrosystem) factors that are associated with self-care. Exclusion criteria included: interventional studies, qualitative studies, reviews (for example, systematic, integrative, etc.) published before 2015, language other than English, and only one self-care behavior as the outcome variable (for example, those just evaluating medication adherence).

### Information sources

The research question and search strategy were developed and reviewed by all authors. In December 2021 four databases were searched; CINAHL Plus with Full Text, Ovid Nursing, PsychINFO, and PubMed. Medical Exact Subject Headings (MeSH) for search terms "heart failure" and "self-care" were entered in each database. Other filters chosen for each database included English language, date (January 1, 2015 to December 31, 2021), peer-review, and abstract available. Results from the four databases were combined, duplicates removed, and final search results were entered into reference management software.

### Study selection

Two authors independently screened a subset of the final search results, evaluating the title and abstract for inclusion. Next, full text of the screened articles were assessed for eligibility by two authors. In this stage, further articles were excluded based on exclusion criteria, discussion, and consensus of the two authors. If needed, a third author participated in the discussion until a consensus was reached. All authors agreed upon the final list of included studies. The research team maintained a list of articles that were included or excluded at each stage of evaluation with a rationale for exclusion. Articles that included secondary analyses were verified to be separate and completely different analyses from the parent study. The final articles for inclusion were entered into Covidence,™, [19] divided, and assigned to two authors for quality assessment.

### Data extraction process

The authors met to discuss and finalize the data that needed to be collected to provide a rich and theory-driven analysis. Each author was assigned an equal number of full-text articles for data collection. Each author entered the individual data in one google sheet. Another author reviewed the google sheet for accuracy and completeness. Data for collection included basic study details (see below) and resultant factors that are positively or negatively associated with self-care.

### Data items

Study details included as data: first author, year of publication, country, theoretical framework, sampling method, setting, sample size, range and mean age of sample, percent male, measurement tools, and the statistical analysis method. Using the review theoretical framework and study findings to identify the summary measures, factors that positively or negatively impact self-care were also collected for this analysis.

### Risk of bias in individual studies- quality assessment

A modified version of The Strengthening the Reporting of Observational Studies in Epidemiology (STROBE) guidelines (STROBE) was entered into Covidence™, [19] (Additional file 1) [20]. Two authors reviewed each article individually against the modified STROBE criteria and assigned a risk of bias using the categories low, high, and unclear that are provided by Covidence systematic review software. Reviewers, as experienced cardiovascular researchers, used their judgment to determine the category for each STROBE criterion. Text from each article substantiating the reviewer’s chosen category was included with their decision. Then the two authors examined the risks of bias to reach a consensus and documented the risk of bias. If the two authors did not agree on the risk of bias a third author would be asked to review and report the bias as well. No articles needed a third reviewer. Data were extracted to an Excel spreadsheet and included authors, year, country, design, theoretical framework, sampling, sample size, setting, instrumentation, statistical analysis methods, mean age, gender, limitations, and results reporting individual and environmental factors.

### Study selection

**Fig. 2:**
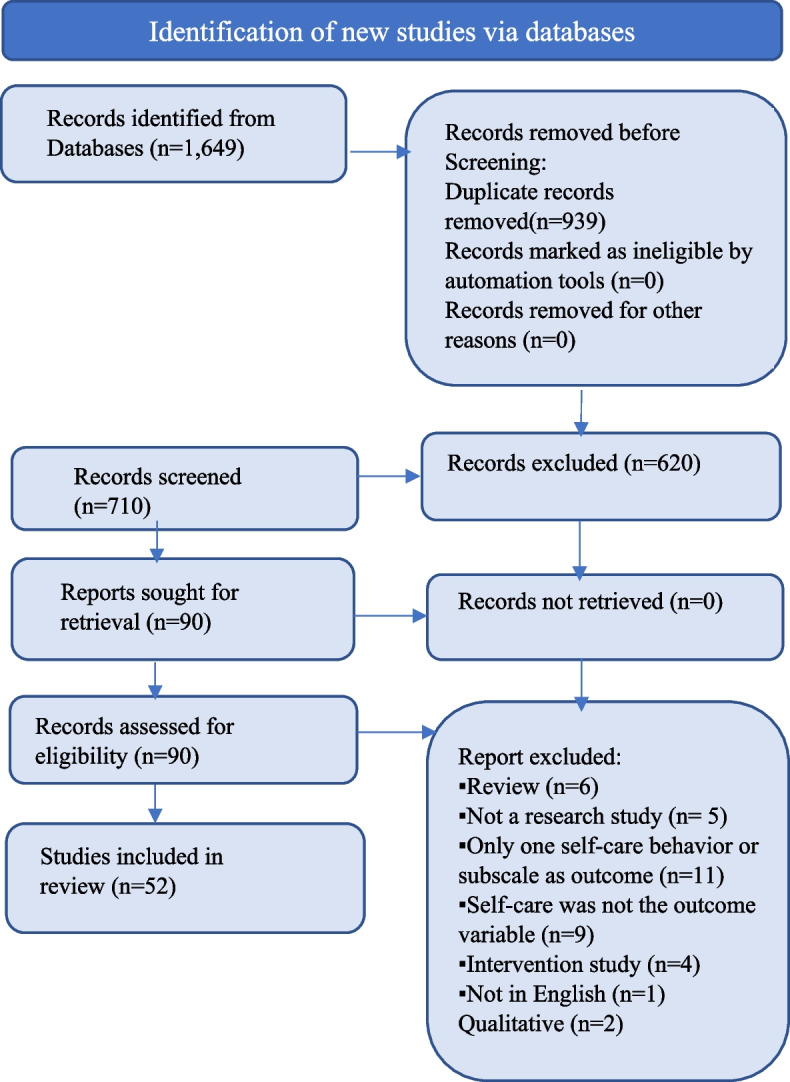
PRISMA diagram Adapted from: Page MJ, McKenzie JE, Bossuyt PM, et al. The PRISMA 2020 statement: an updated guideline for reporting systematic reviews. BMJ. 2021 Mar 29;372:n71. doi:10.1136/bmj.n71. PMID: 33782057; PMCID: PMC8005924 [18]

Studies were identified using the inclusion and exclusion criteria listed from the following four databases: PubMed, Cumulative Index to Nursing and Allied Health Literature (CINAHL) Complete, OVID Medline, and PsychINFO. A total of 1,649 records were reviewed. Once duplicates were removed, 710 record abstracts were screened. Ninety studies were in the initial full-text review; of those, 52 were included in this review. Refer to Fig. 2 Study PRISMA Diagram [18].

### Study characteristics

Of the 52 studies included (see Table 1), the majority were conducted in the United States (*n* = 15, 30%) and were atheoretical (*n* = 36, 70%). The most common theory tested or used to guide the study was the Situation Specific Theory of Heart Failure Self-Care [21] or the revised and updated theory [14]. Convenience sampling was reported in 26% (*n* = 14) of the studies with a large number of secondary analyses (*n* = 15, 28%). The included studies were conducted in many countries, including the United States, Brazil, Italy, Sweden, Germany, Netherlands, Poland, Iran, North Korea, Taiwan, China, Japan, and Ethiopia. Eleven of the 15 studies conducted in the USA included data on participant race. One study from Brazil included race data, and one from Australia identified the country of birth. No other studies included data on race. See Table 1.

**Table 1:** Studies included in the review

Author(s), year and country	Design	Theoretical Framework	Sampling method	Setting	N	Mean sample age (years) M(SD)	% Male	Race	Measurement Tools	Analysis Method
Auld et al., 2018 [22] (USA)	Secondary analysis from longitudinal study	None	Not stated	Community-based outpatient HF clinic associated with academic medical university	146	57 (13.5) No range	70%	None	HFSPS dyspnea, edema; ESS; SCHFI v.6; PHQ9; BSI-anxiety	latent class mixture analysis (LCMA); Latent growth modeling; multivariate backwards stepwise logistic regression
Bidwell et al., 2015 [23] (Italy)	Secondary analysis; cross sectional	Situation specific theory of HF self-care	Not stated	Ambulatory Cardiovascular clinics	364 Dyads	Patient 76.26 (10.7) Caregiver 57.44 (14.6) No range	Patient 57% Informal Caregivers 48%	None	Caregiver-QOL- SF-12; MLHFQ; MMSE; CBI; COPE Index; SCHFI v.6	multilevel modeling; dyadic cross-sectional multivariate outcomes models
Buck et al., 2015 [24] (Italy)	Secondary analysis of cross-sectional database; Self-created model testing	Situation specific theory of HF self-care	Not stated	Cardiovascular centers	628	72.98 (11) No range	57.60%	None	CCI; SCHFI V6.2; MLHFQ	SEM; interaction levels
Bugajski et al., 2021 [25] (Italy)	Secondary Analysis	Situation specific theory of HF self-care	Convenience	Outpatient setting	277 Dyads	Patient: 75.5 (10.8) Caregiver: 52.8 (15) No range	Patient 54.9% Caregiver 29.6%	None	SCHFI 7.2, CC-SCHFI v.2, Dyadic Symptom Management Type (DSMT)	Multilevel Modeling
Cao et al., 2016 [26] (China)	Cross sectional	Situation specific theory of HF self-care	Not stated	In-patient medical wards	127	64.9 (12.34) No range	61.40%	None	SCHFI v.6; DS-14	Multiple linear regression
Cavalcante et al., 2018 [27] (Brazail)	Cross sectional	None	Not stated	In-patient	57	Range 43–95, No mean	57.90%	None	EAAPIC	Student's t-test Mann–Whitney U test
Cené et al., 2015 [28] (USA, North Carolina)	Observational, cross sectional Carolina Data Warehouse for Health (CDW-H) used for eligibility	None	Not stated	Outpatient clinics	150	61 (12) Range 22–84	49%	Black 66 (45%)	SCHFI v.6.2; CES-D; 10-item measure; Blessed test of Orientation-Memory Concentration; Family APGAR	regression analyses with mediation analysis
Chang, et al., 2017 [29] Northern Taiwan	Cross-sectional study	Situation-specific theory of HF	Convenience	Cardiology outpatient clinic	201	62.40 (11.40) No range	69.7%	None	Chinese version SCHFI v. 6.2; Chinese version BDI-II; Chinese version CRS	Moderated mediation model
Choi et al., 2019 [30] (USA, California)	Correlational, cross sectional	Situation-specific theory of HF	Not stated	Cardiomyopathy Center	21	53.8 (7.9) No range	71.4%	None	Demographic data, clinical data from medical record, MoCA, TMT-B, SCHFIv6, MRI	Statistical parametric mapping package, DTI Toolkit, MRIcroN, MATLAB based custom software for images, descriptive statistics, partial correlations using SPSS
Chuang, et al., 2019 [31] (Taiwan)	Cross-sectional study	Situation specific theory of HF (mentioned)	Not stated	Cardiology outpatient clinic	141	65.2 (11.9) Range 26–83	55.3%	None	Chinese version of: PHQ-9; MSPSS; eHealth Literacy Scale; DHFKS; SCHFI v. 6.2	Path analysis
da Conceição et al., 2015 [32] (Brazil)	Descriptive Cross-sectional	a naturalistic decision-making model	Non-probabilistic	Ambulatory care setting	116	57.7 (11.3) Range 20–81	54.30%	White: 69 (59%) Black: 31 (26%) Brown 16 (13.8%)	Brazilian version SCHFI- v 6.2; MMSE; CCI; type of monitoring received by the patient (if supervised exclusively by the physician or by physician and nurse)	ANCOVA model
Davis et al., 2015 [33] (USA)	Secondary analysis, descriptive correlational	naturalistic decision-making process, mentioned	Not stated	In-patient	125	59 (13) Range 22–98	53%	White: 39 (31%) Black: 84 (67%) Other: 2 (2%)	MoCA was SCHFI; DHFKS; GDS; ENRICHD Social Support Inventory; The Charlson Comorbidity Index	Multiple linear regression
Dellafiore et al., 2018 [34] (Italy)	Cross-sectional	none	Convenience sampling	Clinic in a hospital	346	65.6 (13.6) No range	74%	Italian (100%)	SCHFI v..6.2	Logistic regression (LR) models
Dickson et al., 2015 [35] (USA)	Secondary analysis of existing data collected for a prospective cohort study	none	Not stated	Clinics, HF clinic, VA facility	272	> 60 = 53.1% < 60 = 46.9% No range, no mean	62.50%	White: 17(64.7%) Black: 96(35.3%)	MSPSS; SCHFI v.6.2. NART; DHFKS	Adaptive logistic regression. Fisher Exact t-test
Fivecoat et al., 2018 [36] (USA)	Secondary analysis of data from a prospective cohort study	NA	Not stated	Three sites but did not specify	280	61.99(12.47) Range 24–89	64.30%	None	SCHFI v. 6.2	Multilevel modeling, using the SAS PROC MIXED procedure
Freedland et al., 2021 [37] (USA)	Part of a larger study- Not stated	Situation Specific	Not stated	Hospital inpatient	400	58.4 (13) No range	49.5%	49% white	Depression Interview and Structured Hamilton, PHQ-9, SCHFI v.6, Generalized Anxiety Disorder questionnaire, Perceived Stress Scale, Enhancing Recovery in Coronary Heart Disease Social Support Instrument, Kansas City Cardiomyopathy Questionnaire, Duke Activity Status Index	Multi- level modeling
Gebru et al., 2020 [38] (Ethiopia)	Cross-Sectional	None specified	Systematic Random Sampling Technique	Cardiac clinic outpatient clinics	408	45.4 (19) No range	45.1%	None (ethnicity measured)	EHFSCBS, DHKS, MSPSS	Multivariable logistic regression analysis
Graven et al., 2015 [39] (USA) Symptomatology and coping resources predict self-care behaviors	Cross-sectional, correlational predictive	Theory of Stress and Coping	Not stated	Hospital-affiliated outpatient offices in North Florida	201	72.6(8.9) No range	62.70%	Nonminority: 173 (86.1%) Minority: 28 (13.9%)	EHFScBS-9; SPSIR-S; HFSS, Graven and Grant Social Network Survey	Multiple linear regression with true stepwise variable selection was used
Graven et al., 2015 [40] (USA) Predicting depressive symptoms of patients with HF	Cross-sectional descriptive correlational	Stress, Appraisal and Coping Theory	Convenience sampling	Outpatients with HF 3 hospital-affiliated outpatient clinics in Northwest Florida	201	72.57(8.94) Range 55–99	62.60%	White: 173(86.1%) Black: 27(13%) Latino/Hispanic: 1 (5%)	EHFScBS-9; SPSIR-S; HFSS	SEM
Graven et al., 2021 [41] (USA)	Descriptive, cross-sectional, correlational study, secondary analysis	None stated	Not stated	Two acute care facilities	107	61(13.9) No range	54.20%	Non Caucasians: 56%	SCHFI	Multiple linear regression
Heo & Kim, 2020 [42] (Korea)	Cross sectional descriptive	Noe stated	Not stated	Cardiology outpatient clinic	90	72.61 (11.88) No range	36.7%	None	Cardiovascular Disease Resilience Scale CDR, EHFSCB Type D Personality Scale 14	Correlation, hierarchical multiple regression
Heo et al., 2021 [43] (USA)	Cross sectional correlational	None listed	Not stated	HF clinics or hospital units	94	53.6-and 60.3 Two groups of participants, Adherents and non-adherents No range	44%	55% Caucasian	Medication Event Monitoring System, 24 h urine, SCHFI SC management scale, PHQ-9, Control Attitudes Scale-Revised, MSPSS	Logistic regression, Odds ratio, chi square
Hjelm et al., 2015 [44] (Sweden)	Cross sectional	None stated	Not stated	Outpatient clinics at one university hospital and two county hospitals in the south of Sweden	105	***Median*** 72 (65–79)	68%	None	PHQ-9;EHFScBS-9; Word Knowledge test, Neuropsychological test battery, Mini mental state examination, Trail making test	Multiple linear regression
Jo et al., 2020 [45] (South Korea)	Cross sectional-descriptive	None reported	Not stated	Cardiac outpatient clinic	252	73.65(8.08) No range	50.4%	None	BHLS; ESSI (ENRICHD social Support Instrument); EHFScBS-9	Hierarchical regression
Kazeminezhad et al., 2020 [46] (Iran)	Descriptive-analytical	None reported	Not stated	Inpatient	400	Not reported	45.5%	None	Praying Questionnaire Questionnaire of Self-Care behaviors in HF	ANOVA and regression analysis
Kessing et al., 2016 [47] (Netherlands)	Secondary data analysis of longitudinal, baseline 12 and 18 months	None listed	Not stated	Outpatients	545	66.2 (9.6) No range	75%	None	EHFScB-9; FAS; DEFS; HCS; SAD4	SEM
Lee et al., 2017 [48] (USA) Living Arrangements	Secondary analysis of cross-sectional data collected	None listed	Not stated	Ambulatory HF clinics	206	60(11.6) Range 32–87	67%	White: 159 (77.2%) Minority: 47 (22.8%)	SCHFI; PHQ-9; NYHA	Chi Square testing, Spearman correlation coefficient
Lee et al., 2017 [49] (USA) Self-care in rural residents	Secondary Analysis	None listed	Not stated	Rural clinics	508	66 (13) Range 23–96	58.80%	Caucasian: 514 (89%)	EHFScB-9; S-TOFHLA; PHQ-9;. BSI; CAS-R; MLHFQ; Mini-Cog.; NYHA; CCI	Multivariable linear regression
Lee et al., 2019 [50] (Korea)	Cross-sectional, observational	Situation Specific theory of HF self-care	Not stated	Outpatient clinics	132	60(12.8) Range 25–85	72%	None	DHFKS, CAS-R, NYHA, CCI, Seoul Neuropsychological Screening Battery II, Stroop Color/Word Interference Test, backward digit span, and Controlled Oral Word Association Test letter fluency. MOS social support survey	Chi-square test, independent t test, stepwise regression,
Liu et al., 2018 [51] (China)	Secondary analysis. Cross sectional	Mediation conceptual models	Not stated	Cardiac wards in Chinese hospitals	127	64.9(12.34) No range	61.40%	None	SCHFI v 6.2; DS-14D; NYHA	Mediation analysis
Lyons et al., 2017 [52] (USA)	Cross sectional	None listed	Convenience	HF Clinic Pacific Northwest	60 Couples	Patients 59.45 (11.92) Spouses 57.75(11.91) No range	66%	Non-Hispanic White: Patient 88.3% Spouse 86.7%	NYHA; SCHFI v. 6.2; EHFScBS-9;	Multilevel modeling
Massouh et al., 2020a [53] (Lebanon) Determinants	Cross-sectional correlational	Self-care in chronic illness theory	Consecutive	Inpatient units and outpatient units at a tertiary medical center	*100 (51 respondents to Self-Care Management scale)	67.59 (12.09) No range	76%	None	SCHFI (Arabic version), NYHA, CCI, PHQ-9, ESSI, DHFKS	Descriptive statistics, independent t test, ANOVA or Pearson R, regression
Massouh et al., 2020b [54] (Lebanon) Self-care confidence	Cross-sectional, correlational	None noted	Consecutive	Inpatient units and outpatient units at a tertiary medical center	100	67.59 (12.09) No range	76%	None	SCHFI (Arabic version), ESSI, DHFKS	Descriptive statistics, independent t-test, ANOVA or Pearson r, mediation analysis,
Masterson-Creber et al. 2017 [55] (USA)	Prospective cross-sectional	None listed	Not stated	Academic urban medical center	96	56.9 (12.4) Range 23–77	65%	White: 28% Black: 39% Other: 28% Asian: 4%	PAM–13; EHFSC-9; SCHFI v 6.2; Control and Attitudes Scale. Kansas City Cardiomyopathy. 3 health literacy questions; Heart Failure Somatic Perception Scale. Patient-Reported Outcomes Measurement Information System short-form; Physical Function, Depression, Anxiety, Fatigue, Applied Cognition, and Sleep Disturbance;	Fisher Exact
Matsuoka et al., 2016 [56] (Japan)	Cross-sectional, observational study	None listed	Not stated	Academic and Rural Hospitals	227	67.7 (13.9) No range	62.6%	None	Japanese version: EHFScBS-9; Health Literacy Scale; NYHA	Multi-variate linear regression analysis
Moaddab et al., 2020 [57] (Iran)	Cross sectional, descriptive	None listed	Convenience	Inpatient and outpatient clinic at referral hospital	239	59.04 (9.91) No range	68.6%	None	SCHFI, CDS, MMSE,CCI	Descriptive statistics including Spearman correlation, Chi square, Fisher's exact test, Mann Whitney, Kruskal Wallis and logistic regression
Muller-Tasch et al., 2018 [58] (Germany)	Cross sectional	None listed	Consecutive	Cardiac outpatient units	308	63.6 (12.1) Range 19–90	74.70%	None	PHQ- 9; EHFScB	Multivariate analysis
Nadrian et al., 2018 [59] (Iran)	Secondary analysis. Prospective experimental study	Health Belief Model	Not stated	Heart Hospital	180	53.2 (12.5) Range 20–79	79.80%	None	Do not list tools used/secondary analysis	Regression path analysis
Nesbitt et al., 2021 [60] (Australia)	Descriptive correlational	none	Not stated	Public outpatient HF clinics	36	67.5 (11.3) No range	80.6%	Identified country of birth	General literacy, Rapid estimate of adult literacy in medicine short -form, Short test of functional health literacy in adults, SCHFI, DHFKS	Descriptive statistics using mean, median, IQR, categorical using proportions, correlations Spearman's rho
Ok et al., 2015 [61] (Korea)	Correlational	NA	Consecutive	Participants were patients with HF who visited the outpatient cardiology clinics of three tertiary hospitals in a metropolitan area in Korea	280	59.5 (13.83) No range	65% (182)	None	EHFScBS-9; Duke Activity Status Index; DHFKS, NYHA; MOS- Social Support	Multiple regression analysis
Park et al., 2020 [62] (South Korea)	Descriptive, cross sectional	None noted	Convenience	Outpatient	170	67.09 (12.02) No range	62.4%	None	Sociodemographic characteristics, DS-14, EHFScBS-9 Korean version,	Descriptive statistics, independent t test, one way ANOVA, point by serial correlation Pearson correlation, mediation analysis using PROCESS
Prochota et al., 2019 [63] (Poland)	Prospective, observational	None noted	Not stated	Internal medicine department of the health care center in Oleśno	100	73.78 (8.98) Range 60–88	52%	None	Sociodemographic characteristics, EHFScBS-9, MMSE	Descriptive statistics including percentages and counts for qualitative, Student's t-test, Mann Whitney, ANOVA, Kruskal–Wallis, with post-hoc analyses and Bonferroni correction
Seid et al., 2019 [64] (Ethiopia)	Cross sectional	None noted	Not stated	Outpatient clinic at a referral hospital	310	49(19.5) Range 18–89	35.8%	None	Revised Heart Failure Compliance Scale; JHFKS	Logistic regression
Siabani et al., 2016 [65] (Iran)	Cross sectional	NA	Not stated	Inpatient	255	66 (13) No range	(51.5%) 119	None	Persian version: SCHFI	Univariate analysis, multiple linear regression analysis
Son et al., 2018 [66] (South Korea)	Cross sectional	None noted	Convenience	Outpatient clinical at a general hospital	281	68.7(11.1) No range	60.9%	None	Korean version: of the Frail scale, EHFScBS-9	Independent t test, one way ANOVA, Pearson's correlations, and hierarchical regression
Uchmanowicz et al 2015 [67] (Poland)	No mention of design but it is cross sectional	None noted	Not stated	Cardiology clinic	110	66.1(11.4) No range	53.64%	None	Polish version: Tilburg Frailty Indicator; EHFScBS-9	Pearson's, Spearman's rho correlations, stepwise regression
Uchmanowicz et al., 2017 [68] (Poland)	Cross sectional	None noted	Not stated	Cardiology clinic	270	72.57(8.23) No range	48.89%	None	MMSE; EHFScBS-9	ANOVA
Vellone et al., 2015 [69] (Italy)	Secondary analysis-mention cross sectional in the limitations section	situation specific theory of HF self-care	Not stated	39 CV ambulatory clinics in 29 provinces in Italy	628	73 (11) No range	58%	None	SCHFI v 6.2; MMSE; CCI	SEM
Vellone et al., 2017 [70] (Italy)	Secondary analysis	none reported	Not stated	Cardiovascular outpatient clinics	1192	72.4 (11.2) No range	58%	None	EHFScBS-9; MMSE, Barthel Index; CCI; QOL SF 12; MLHFQ	ANOVA
Wang et al., 2020 [71] (China)	Cross sectional	None reported	Convenience	Hospital	310	68.62 (13.39) No range	53.2%	None	Sociodemographic characteristics, clinical characteristics, HADS Chinese version, Health Literacy Scale Chinese version, SCHFI-Chines version	Descriptive statistics, correlation, mediation using PROCESS,
Yang & Kang, 2018 [72] (Korea)	SEM	Theory of Unpleasant symptoms	Convenience	Outpatient clinics	209	67.71 Range 28–89	46.4% male	None	NYHA; Korean version: HADS; Inventory of social supportive behaviors; MSAS-HF; SCHFI	SEM
Zou et al., 2017 [73] (China)	Cross sectional	They did use a conceptual model to guide the study see pg. 531	Convenience	Three CV units from a large general university hospital in one province Shandong	321	63.6 (10.6) No range	51.4%	None	Chinese version: SCHFI DASI, HFKT-Ct, Health Literacy Scale for Patients with Chronic Disease; MSPSS; MSSSS	SEM and mediation analysis

The majority of studies had mean ages of 50 and older with only two [38, 64] reporting a mean age of less than 50 and some not reporting means at all. Both studies with a lower mean were conducted in Ethiopia, possibly indicating a younger age of HF disease. The majority of settings were inpatient or outpatient HF clinics. Most studies (*n* = 31, 60%) recruited from and/or conducted their research in outpatient clinics, while 25% (*n* = 13) used inpatient sites. Four studies recruited from inpatient and outpatient settings (7.5%), while four others did not indicate or were unclear where participants were recruited from (7.5%). No studies were conducted in home settings or general cardiology practice, but many were conducted in outpatient HF or cardiology clinics. The number of participants ranged from 21–1192, with a total for all included studies of 12,709 participants (individuals or dyads) with a mean sample size of 246. The Self-Care in Heart Failure Index (SCHFI) (*n* = 30, 58%) and the European Heart Failure Self-Care Behaviour Scale (EHFScBS-9) (*n* = 17, 32%) were the two most commonly cited self-care measurement tools in the included research studies (See Table 2 for abbreviation key). Most studies (*n* = 43, 83%) had a majority of male participants. Only eight studies (15%) had less than 50% male participants. One study was 50% male, with a mean percentage of male participants across all studies of 63.1%. See Table 2 for abbreviation explanations.

**Table 2:** Key of abbreviations

Full name	Abbreviation
Adult Reading Test	NART
Beck Depression Inventory Second Edition	BDI-II
Brain-natriuretic peptide	BNP
Brief Health Literacy Scale	BHLS
Brief Symptom Inventory	BSI
Careers of Older People in Europe Index	COPE Index
Caregiver Burden Inventory	CBI
Center for Epidemiologic Studies Depression	CES-D
Charlson Comorbidity Index	CCI
Chinese version of the Resilience Scale	CRS
Control Attitude Scale Revised	CAS-R
Duke Activity Status Index	DASI
Dutch Exertion Fatigue Scale	DEFS
Dutch Heart Failure Knowledge Scale	DHFKS
Enhancing Recovery in Coronary Heart Disease	ENRICHD
ENRICHD Social Support Instrument	ESSI
Epworth Sleepiness Scale	ESS
Family Adaptability Partnership Growth Affection and Resolve	Family APGAR
Fatigue Assessment Scale	FAS
Geriatric Depression Scale	GDS
Glomerular Filtration Rate	GFR
Hospital Anxiety and Depression Scale	HADS
Heart Failure Somatic Perception Scale	HFSPS
Heart Failure Symptom Survey	HFSS
Heart Failure Knowledge Test-Chinese version	HFKT-C
Japanese Heart Failure Knowledge Scale	JHFKS
Macarthur Scale of Subjective Social Status	MSSSS
Medical Outcomes Study	MOS
Memorial Symptom Assessment Scale-Heart Failure	MSAS-HF
Mini-Mental State Examination (MMSE) Careers of Older People in Europe Index (COPE Index);	MMSE
Minnesota Living with Heart Failure Questionnaire	MLHFQ
Montreal Cognitive Assessment	MoCA
Multidimensional Scale of Perceived Social Support	MSPSS
New York Heart Association	NYHA
Patient Activation Measure	PAM
Patient Health Questionnaire	PHQ9
Scale of Evaluation of the Self-care of Patients with Heart Failure	EAAPIC
Self-care of HF Index	SCHFI
Short Form-12	SF12
Social Problem Solving Inventory Revised-Short	SPSIR-S
Symptoms of Anxiety-Depression Index	SAD4
The European Heart Failure Self-care Behavior Scale 9	EHFScBS-9
Type D personality scale	DS-14

### Risk of bias within studies

**Fig. 3:**
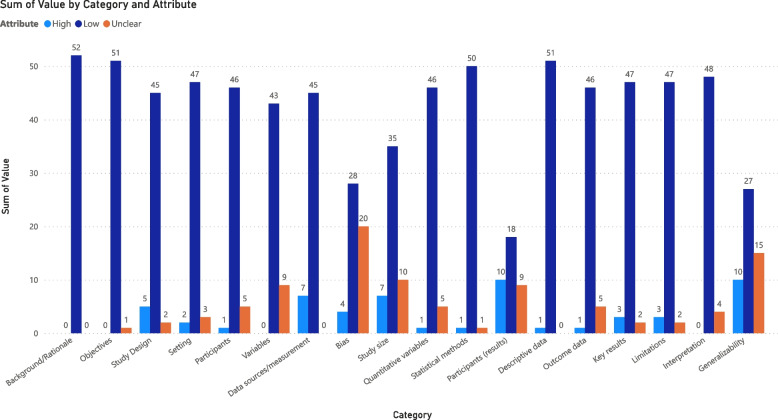
Risk of bias in included studies

All articles were assessed for bias and categorized as either low, high, or unclear for each category of potential bias (see Fig. 3 Bias Bar Chart). Results of the quality assessment revealed that on the criteria of background/rationale, objectives, participants, variables, statistical methods, descriptive data, outcome data, and interpretation for the study, overall, authors were clear and thoroughly addressed the modified STROBE criteria. The criterion of generalizability and participants (results) scored highest (*n*= 10). In these studies, participant numbers at each stage and/or generalizability were not addressed. Most articles had low percentages, with 80% or more assessed as low bias across all criteria, except for bias, where 20 (38%) were rated as unclear and 4 (1%) as high risk of bias. This reflects a deficit in describing efforts made to address potential sources of bias [16].

### Risk of bias across studies

The risk of bias across studies in this review is related to self-report measures. Most of the studies used self-reported survey results for analysis. Social desirability, sampling, and recall bias are risks associated with self-report [74]. Most surveys used in the included studies were previously validated and considered reliable, thus reducing the risk of social desirability. If concerns remain about social desirability, it can be measured with the Marlowe–Crowne Social Desirability Scale [75] or Martin–Larsen Approval Motivation score [76]. None of the studies reported measuring social desirability.

Sampling bias is inherent in convenience samples, which most studies employed. Ways to correct for sampling bias include matching the sampling frame with the target population, making surveys short and accessible, non-responder follow-up, or oversampling. Most studies did not address sampling bias in the ways mentioned above [77]. Recall bias was a reduced risk in the included studies because the questions predominantly pertained to routine or frequent events and the study designs are prospective. Although, there is evidence that demographic variables, such as the diagnosis of HF, are associated with recall bias [78]. This may result from a decrease in cognitive function experienced by those with HF. Data can be corroborated with direct observation, case–control studies, or use of biological markers [79, 80]. None of the included studies addressed the potential for recall bias.

The results of the individual studies were divided according to the level at which the factors fit within the levels of the adapted socioecological model. Studies that investigated or reported complex relationships are discussed as well.

### Individual level factors

Overwhelmingly the literature is focused on individual-level factors that impact an individual's ability to provide self-care. Very few studies examined system-level factors. There were more positive associations with self-care than negative associations. When reporting an association between variables, it indicates there is some sort of relationship between the two variables. If there is a positive association when one of the variables increases the other variable increases. If there is a negative association when one of the variables increases the other variable decreases. Specific levels of association (correlation) are not included in this review and can be found in the original version of each included article. Some variables were dichotomous or categorical, for example, sex (male/female) so that when reported, the association is of the specific subcategory and self-care.

The positive associations most often cited were self-care efficacy/self-care confidence [24, 31, 34, 53, 69, 72], HF knowledge [38, 53, 59, 61, 64], health literacy [45, 56, 66, 71], and symptoms [22, 39, 40]. Auld et al. [22] reported that a high number of symptoms sustained over long periods are positively associated with self-care. Additional demographic factors that were positively associated with self-care included marital status [27], higher household income [27], male gender [64], education level [22, 47, 66, 68], female gender [52], being a minority [39], unemployment [47] and having diabetes [47]. Additional factors that were positively associated included higher activation levels [55], higher cognitive function [57], having a religion and prayer [42, 46], and perceptions about the barriers to self -care [59] and perceived risk (susceptibility) [37].

Some factors were negatively associated with self-care. Age [38, 63, 68], cognitive impairment [23, 33], higher NYHA class [38, 39, 63, 68], and depressive symptoms [37, 49, 58, 71] were the top reported factors negatively associated with self-care. Lee et al. [48] found that depressive symptoms influenced those living alone more than those living with someone else. Additional cognitive function abilities were investigated and reported by Hjelm et al. [44] who found that poor executive function and poor psychomotor speed were negatively associated with HF self-care. Some findings described as negative, confirmed positive associations. For instance, Dellafiore et al. [34] reported that *inadequate* self-care confidence was negatively associated with self-care, consistent with the findings that self-care confidence and self-efficacy are associated with good self-care. Symptoms also played a role in the negative associations with low symptoms that were sustained [22], more and unpleasant symptoms [72], general fatigue [47], or experiencing HF symptoms in general [40]. Some demographic and clinical factors associated with lower levels of self-care included lower Left Ventricular Ejection Fraction (LVEF) [32], and having HF a long time [68]. Contradictory findings reported that symptoms can motivate one to become more engaged in self-care behaviors (a positive relationship) or can have the opposite effect and can potentially hinder engagement in self-care (a negative relationship).

### System level factors

System-level factors are those that are associated with the microsystem, exosystem, and macrosystem levels of Bronfenbrenner's [17] Socioecological Model. System factors are outside the individual and include family, peers, friends, extended family, neighborhood (micro), work environment, mass media, healthcare organizations, social organizations, religious organizations (exo), laws, culture, history, social conditions, and the economic system (macro). The results of this systematic review demonstrate that the vast majority, more than 35, factors reported are at the individual level with only 11 micro-level factors measured associated with self-care. Micro-system level factors that have a positive relationship with self-care included: caregiver relationship quality [23], marital status [27], household income [27], people accompanying patients to visits [28], and social support [28, 38, 40, 45, 50, 61, 62, 67]. Some authors examined specific dynamics within the broader context of social support. Bidwell et al. [23] found that caregiver relationship quality was important to self-care, while Cene ´et al. [28] noted that when people with HF had someone accompany them to healthcare visits, they performed better self-care. Similarly, Graven et al. [39] found that individuals who had higher social network scores (number of people who provide assistance and support and their satisfaction with the support provided) had better self-care. In contrast, Lyons et al. [52] reported that average confidence level in the dyad (patients and spouses/partners) was associated with engagement in self-maintenance, self-management, and consulting. Living alone [48, 68] and lack of a partner [47] are negatively associated with self-care. System-level factors tend to be more complex than individual factors to measure and can be more difficult to capture and determine which components of various policies or programs contributed to the change in self-care. No studies were reviewed that measured factors at the exosystem or macrosystem levels.

### Complex relationships

Numerous studies demonstrated complex relationships between variables that impact self-care. Most of these studies used path analysis or structural equation modeling for statistical analysis [24, 25, 29, 40, 47, 51, 52, 59, 69, 72, 73]. Self-care confidence was measured frequently. The terms self-care confidence and self-efficacy were used interchangeably by researchers using the same measurement tool (SCHFI self-efficacy scale). Self-care confidence was considered a mediator between cognition [69], depression [72], and moderate or high resilience [29] on self-care maintenance. Self-care confidence also mediated the relationship between social support and self-management [72]. Massouh et al. [54] found that self-care confidence mediated the relationship between social support and self-maintenance and the relationship between HF knowledge and self-maintenance and self-management. Vellone et al. [69] found that self-care confidence mediated the relationship between cognition and self-management and cognition and self-maintenance. Self-care confidence mediated the relationship between negative affectivity and social inhibition with self-care maintenance [51]. Symptom perception is associated with congruence in HF dyads; the better the dyad congruence, the better the symptom perception [25]. Unpleasant symptoms mediated the relationships between disease severity, anxiety, and self-care [72]. Symptom severity mediated social support and self-care [39]. Knowledge, perceived susceptibility, and perceived barriers mediated the relationships between locus of control, perceived severity, perceived threat, perceived benefits, cues to action, and self-care [59]. Vellone et al. [70] discovered when looking between three clusters of HF patients that the cluster of patients with the best self-care included those who are younger, have higher education, high levels of employment, higher income, shorter illness duration, higher EF, NYHA class I and II, lowest number of medications, low BNP level, can perform more ADLs, have the highest cognition levels, the best specific physical QOL, and have lower hospitalization rates. The other three clusters identified had low to inconsistent adherence. Prefrontal brain tissue integrity (responsible for memory, problem-solving, and decision-making), measured via MRI, has a positive relationship with self-care [30].

### Summary of evidence

Self-care in HF is a widely studied concept in many different countries. This can be attributed to the number of people globally suffering from HF and the fact that the two most used tools to measure self-care are freely available (the SCHFI and EHFScBS-9). In six studies, self-confidence or self-efficacy was found to be associated with self-care [24, 31, 34, 53, 69, 72]. HF knowledge [38, 53, 59, 61, 64], and general education level were associated with self-care [22, 38, 47, 66, 68]. Depressive symptoms related to self-care in seven studies [37, 49, 58, 71]. Two studies found cognitive impairment predicted self-care [23, 33]. Social support was also found to impact self-care in seven studies [28, 38, 40, 45, 61, 62, 67].

The SCHFI v6.2 includes three scales: self-care maintenance, self-care management, and self-efficacy. Therefore, when using this instrument, not only could self-care as a singular concept be measured but relationships between factors and the three scales could also be described. For example, minority status [33, 39, 41] and emotional quality of life was associated with self-care maintenance [23, 24]. General education level was found to impact self-management [33, 43, 65]. See Table 3. Many other individual factors and some microsystem factors were associated with self-care. Many researchers used the SCHFI tool to measure self-care behaviors and self-efficacy. If a factor was associated with self-management and self-maintenance, it was included as being associated with self-care. The second most used tool for measuring HF self-care was the EHFScBS-9. This is a one-factor tool with no subscales. It does not separate between self-management and self-maintenance behaviors.

**Table 3:** Relationship between factors and self-care

Direction of relationship	Individual Level Factors	Microsystem Level Factors
Self-care positive	Activation [55]	Caregiver relationship quality [23]
	Cognitive function [57]	Confidence in the dyad [52]
	Coping (problem solving) [39]	Household income [27]
	Diabetes mellitus [47]	Joint monitoring by nurse and physician [32]
	Education Level [22, 38, 47, 66, 68]	Marital status [27]
	Executive function [43]	People accompanied patients to visits some or most every visit [28]
	Frequency of HF symptoms [39]	Social component of Frailty Syndrome (decreased risk for social isolation) [67]
	Having a Religion [42]	Social network [39]
	Health literacy [45, 56, 66, 71]	Social Support [28, 38, 40, 45, 61, 62, 67]
	HF knowledge [38, 53, 59, 61, 64]	
	History of receiving information [57]	
	Income [27, 57]	
	Length of time with physical symptoms, sustained symptoms- over 6 months [22]	
	Length of time with HF [32]	
	Lower functional status [61]	
	LVEF [63, 68]	
	Medical aid [42]	
	Minority [39]	
	No occupation [42]	
	Non-Type D personality [42, 67]	
	Number of comorbidities [3, 8]	
	Perceived control [50]	
	Perceived barriers to self-care [58]	
	Perceived susceptibility (risk) [59]	
	Praying [46]	
	Prefrontal brain tissue integrity [30]	
	Psychomotor speed [43]	
	Self-care confidence/ Self-efficacy [24, 31, 34, 53, 69, 72]	
	Sex Females [52]	
	Sex Males [64]	
	Social problem solving [40]	
	Symptom-related interference with enjoyment of life [40]	
	Unemployed [47]	
Self-care negative	Age [38, 63, 68]	
	Anxiety [58]	Lack of a partner [47]
	Cognitive impairment [23, 33]	Living alone [48, 68]
	Comorbidity [64]	Patients when compared to informal caregivers were more engaged [52]
	Depressive symptoms [37, 49, 58, 71]	
	Disease severity [33]	
	General Fatigue [47]	
	Hospital readmission [49]	
	Length of time diagnosed with HF [68]	
	LVEF [32]	
	Minority status [35]	
	NYHA classification [38, 39, 63, 68]	
	Perceived barriers to self-care [55]	
	Physical symptoms [39]	
	Sex- Male [47]	
	Symptom status [49]	
	Type D personality [26, 42]	

## Discussion

The purpose of this review was to establish the socioecological levels of current research in HF self-care. It is clear that confidence in one's ability to carry out self-care behaviors and education (both HF and general) is frequently associated with the level of self-care one performs, as does depressive symptoms, cognitive dysfunction, and social support. Self-efficacy, cognitive health, HF education, social support, and preventing or treating depressive symptoms are areas for intervention development. Many patients have a combination of negative factors that may be related to their ability to self-care. Between 25–75% of people with HF experience cognitive impairment [81], and up to 33% experience depressive symptoms, with 19% meeting the criteria for a diagnosis of depression [82]. Both cognitive impairment and depressive symptoms have been related to adverse outcomes, including difficulties in self-care [23, 33, 37, 49, 58, 71]. Factors that impact self-care negatively can intersect, making the readiness and ability to self-care more difficult. Many other factors negatively or positively impact HF self-care, as shown in Table 3. Positive factors may provide a protective effect while negative factors may have a harmful effect. Having an idea of how many and to what degree an individual experiences the negative and positive factors related to self-care could guide personalized interventions that would provide more nuanced, more person-centered treatment.

What is evident from this systematic review is that there were no exosystem or macrosystem factors measured in the included articles. Therefore, there is a void in research measuring system-level factors that may impact self-care in people with HF. If we are to extend and grow HF self-care science, factors that impact HF self-care at the systems levels and their interactions need to be investigated [83]. According to Kindig & Isham [84] individual behaviors account for 30% of health outcomes, and 20% are due to clinical care. The remaining 50% of health outcomes are derived from social and economic determinants of health (40%) and physical environment (10%). Therefore, it is crucial to capture factors at all levels of the socioecological model that may impact HF self-care. Examples of factors in the exo- and macrosystem are home health services (what is offered, who is receiving these services, and are there HF home health protocols that could provide consistent guidelines for home care of those with HF), geographic information systems (GIS) hotspot areas with high-density HF to relocate services, healthcare mistrust, access to care, organizational literacy, provider cultural competency, economic stability, housing, transportation, access to walkable and safe areas, access to healthy eating options, healthcare system policies, institutional racism, access to insurance and medication.

### Limitations of included articles

Definitions of self-care were not always clear and consistent. Articles did not consistently report research using well-established guidelines. A common observation of the reviewed studies was not defining or reporting the sampling method. Many studies did not identify the sampling method as convenience, although based on the other information given in the participant sections of the papers, it was evident convenience sampling was most likely employed. This could be related to using secondary analyses as the study design (the reader was directed to the parent study) but not in all cases. Study size estimations with power analyses were not included in many articles. Concepts being measured were not defined theoretically- they were defined operationally using specific tools/measures. Not defining the terms theoretically can make comparisons more difficult although two main measurement tools were used throughout the studies- the SCHFI v6.2 and EHFScBS-9. Another outcome level limitation was that there were many countries represented where people may have differing perceptions and resources available. This was discussed in numerous articles as a limitation of study generalizability. Yet this could also be seen as an advantage with multiple diversities represented. Many of the included studies did not address the risk of bias and ways used to mitigate it. Also, many articles were missing participant attrition (using a Consort diagram or narrative). Missing data and how that was managed was also an area of weakness. Publishing requirements may also limit the information in articles, which may present another limitation. Some studies are not published; thus, perhaps valuable information is unavailable when trying to fully describe the factors associated with HF self-care.

### Limitations of this systematic review

The major limitation of this systematic review was that it was not a complete review of all relevant HF self-care research over an extended length of time. HF self-care quantitative research has been prolific since the two tools, SCHFI and EHFScBS-9, were made available. This systematic review itself did not consider qualitative studies or intervention studies. A complete picture of HF self-care can be discerned with a comprehensive review of all three types of studies. Other older and methodologically diverse HF self-care literature reviews can be accessed to develop a more complete picture of HF self-care research over time [10–13].

## Conclusions

Nurse researchers need to capture and measure the social determinants of health that impact self-care. Measuring these determinants will move science to the outer levels of the socioecological model. Researchers do not need to forgo measuring individual health behaviors, but balancing the factors analyzed would provide a more complete picture of the factors impacting self-care and enable us to help patients achieve their goals. In healthcare safety we try to maintain a just culture that acknowledges the system-related contributors to safety as being primary. We need to do the same with self-care. Measuring system-related factors will help "grow the evidence base" [83]. Describing and evaluating these system-level factors can be difficult because there are many aspects to them, the science may need to adopt a program evaluation methodology. If we want to enhance the health of people with HF, we will have to address how to keep people as healthy as possible, balancing the interest in how individual behaviors potentially influence self-care with the effectiveness of policies and systems that impact self-care.

### Supplementary Information

**Additional file 1.**

## Data Availability

The datasets generated and/or analyzed during the current study are available in CINAHL Plus with Full Text, Ovid Nursing, PsychINFO, and PubMed databases.
