# Supplementary material for: Individual and systems-related factors associated with heart failure self-care: a systematic review
Source: BMC Nurs. 2024 Feb 9;23:110. doi: 10.1186/s12912-023-01689-9 (PMC10854154; doi:10.1186/s12912-023-01689-9)
Supplement: Supplementary file 1 — Additional file 1. [file 12912_2023_1689_MOESM1_ESM.docx]

Online Appendix A

| Modified STROBE | |
| --- | --- |
| Background/rationale | Explain the scientific background and rationale for the investigation being reported |
| Objectives | State specific objectives, including any pre-specified hypotheses |
| Study design | Present key elements of study design early in the paper |
| Setting | Describe the setting, locations, and relevant dates, including periods of recruitment, exposure, follow-up, and data collection |
| Participants | Give the eligibility criteria, and the sources and methods of selection of participants |
| Variables | Clearly define all outcomes, exposures, predictors, potential confounders, and effect modifiers. Give diagnostic criteria, if applicable |
| Data Sources/measurement | For each variable of interest, give sources of data and details of methods of assessment (measurement). Describe comparability of assessment methods if there is more than one group RELIABILITY- Cronbach's reported |
| Bias | Describe any efforts to address potential sources of bias |
| Study size | Explain how the study size was arrived at |
| Quantitative  variables | Explain how quantitative variables were handled in the analyses. If applicable, describe which groupings were chosen and why |
| Statistical  methods | (1) Describe all statistical methods, including those used to control for confounding  (2) Explain how missing data were addressed |
| Participants | Report numbers of individuals at each stage of study—eg numbers potentially eligible, examined for eligibility, confirmed eligible, included in the study, completing follow-up, and analysed |
| Descriptive  data | Give characteristics of study participants (eg demographic, clinical, social) and information on exposures and potential confounders |
| Outcome  data | Report numbers of outcome events or summary measures- the outcome of the statistical analysis |
| Key results | Summarize key results with reference to study objectives |
| Limitations | Discuss limitations of the study, taking into account sources of potential bias or imprecision. Discuss both direction and magnitude of any potential bias |
| Interpretation | Give a cautious overall interpretation of results considering objectives, limitations, multiplicity of analyses, results from similar studies, and other relevant evidence |
| Generalizability | Discuss the generalizability (external validity) of the study results |
